# CO_2_-induced ocean acidification does not affect individual or group behaviour in a temperate damselfish

**DOI:** 10.1098/rsos.170283

**Published:** 2017-07-05

**Authors:** Garfield Tsz Kwan, Trevor James Hamilton, Martin Tresguerres

**Affiliations:** 1 Marine Biology Research Division, Scripps Institution of Oceanography, University of California San Diego, 9500 Gilman Drive, La Jolla, CA 92093, USA; 2 Department of Psychology, MacEwan University, Edmonton, Alberta, Canada T5 J 4S2; 3 Neuroscience and Mental Health Institute, University of Alberta, Edmonton, Alberta, Canada T6G 2H7

**Keywords:** climate change, upwelling, GABA, blacksmith, anxiety, shoaling

## Abstract

Open ocean surface CO_2_ levels are projected to reach approximately 800 µatm, and ocean pH to decrease by approximately 0.3 units by the year 2100 due to anthropogenic CO_2_ emissions and the subsequent process of ocean acidification (OA). When exposed to these CO_2_/pH values, several fish species display abnormal behaviour in laboratory tests, an effect proposed to be linked to altered neuronal GABA_A­_ receptor function. Juvenile blacksmith (*Chromis punctipinnis*) are social fish that regularly experience CO_2_/pH fluctuations through kelp forest diurnal primary production and upwelling events, so we hypothesized that they might be resilient to OA. Blacksmiths were exposed to control conditions (pH ∼ 7.92; *p*CO_2_ ∼ 540 µatm), constant acidification (pH ∼ 7.71; *p*CO_2_ ∼ 921 µatm) and oscillating acidification (pH ∼ 7.91, *p*CO_2_ ∼ 560 µatm (day), pH ∼ 7.70, *p*CO_2_ ∼ 955 µatm (night)), and caught and tested in two seasons of the year when the ocean temperature was different: winter (16.5 ± 0.1°C) and summer (23.1 ± 0.1°C). Neither constant nor oscillating CO_2_-induced acidification affected blacksmith individual light/dark preference, inter-individual distance in a shoal or the shoal's response to a novel object, suggesting that blacksmiths are tolerant to projected future OA conditions. However, blacksmiths tested during the winter demonstrated significantly higher dark preference in the individual light/dark preference test, thus confirming season and/or water temperature as relevant factors to consider in behavioural tests.

## 1. Introduction

Atmospheric carbon dioxide (CO_2_) is rapidly increasing due to burning of fossil fuels, cement production and land use changes [1]. Since the industrial revolution, the ocean has absorbed approximately 30% of atmospheric CO_2_, which has raised CO_2_ levels in the surface ocean from approximately 280 to approximately 400 µatm [2,3]. This has resulted in a reduction in ocean pH and a shift in carbonate chemistry, a process called ocean acidification (OA) [2–4]. The current global surface ocean pH is already 0.1 units lower than the pre-industrial level, and the Intergovernmental Panel on Climate Change's IS92a ‘business as usual’ projection predicts a further CO_2_ increase to approximately 800 µatm and an associated pH drop of 0.3–0.4 units by the year 2100 [4,5].

Numerous laboratory studies have found behavioural changes in response to future OA conditions including improper reaction to predator, prey, parental and habitat cues [6–8]. Studies have also reported decreases [9,10] and increases [11] in behavioural lateralization, impaired learning [12,13], increased anxiety-like behaviour [14] and increased boldness [15] (reviewed in [16]). Field-based OA experiments have additionally documented impaired predatory cue detection [7,17,18], homing ability [19] and habitat preference [20]. Many of those effects seem to be at least partially due to altered movement of Cl^−^ ions through γ-aminobutyric acid type A (GABA_A_) receptors, because administration of the GABA_A_ antagonist gabazine restored the discrimination of olfactory cues [21], behavioural lateralization [9,10] and learning [12] (reviewed in [22]). Additionally, the application of gabazine induced anxiety-like behaviour in control fish to levels similar to fish exposed to OA conditions [14]. However, some other studies have reported a lack of effect of OA-like conditions on fish subjected to tests on behavioural lateralization, emergence from shelter, predator odour avoidance, foraging and anxiety-like behaviour [23–26], suggesting fish species-specific responses to OA.

GABA_A_ receptors are the major inhibitory mechanism in the central nervous system of vertebrate animals, including marine fish [27,28]. A widespread approach to examining whether GABA_A­_ receptors are functioning abnormally is through the testing of anxiety-like behaviour. These tests are based on evidence that activation of GABA_A­_ receptors inhibits neural excitability, thus causing a reduction in anxiety; conversely, anxiety is potentiated by GABA_A_ receptor antagonist drugs [29,30]. The light/dark preference test is a common test for anxiety-like behaviour in mice [31,32] and zebrafish [33], and has recently been used also in other fish species [14,25,34,35]. In the light/dark preference test, an organism is placed in a rectangular arena containing walls that are white on one-half of the arena and dark on the other half. An anxious organism will have a tendency to seek the dark side of the arena to hide from aversive stimuli, while a less anxious organism typically explores the light side of the arena. The administration of pharmacological compounds can have a significant impact on the behaviour of the organism in the light/dark preference test. For example, the GABA_A­_ agonists diazepam, clonazepam, bromazepam and chlordiazepoxide decreased the time spent in the dark zone in zebrafish [36,37]. Conversely, GABA_A­_ antagonists such as picrotoxin and gabazine increased the time spent in the dark zone, and therefore anxiety, in mice [38], zebrafish [39] and splitnose rockfish (*Sebastes diploproa*) [14]. As OA is proposed to alter GABA_A­_ receptor functioning [21,40,41], tests of anxiety-like behaviour like the light/dark preference test are well suited to investigate this potential mechanism of physiological change [14].

To date, most OA studies have investigated the effects of elevated CO_2_-induced acidification based on global-scale surface ocean predictions, and have acclimated fish to constantly elevated CO_2_ (and thereby reduced pH) levels. However, this situation does not adequately represent the large, natural variability of coastal environments caused by near shore processes such as upwelling, water advection and primary production [42–45]. Furthermore, in some instances, coastal CO_2_/pH changes can exceed the predicted pH changes of the global surface ocean [45]. For example, in the La Jolla kelp forest (San Diego, USA), pH at 7 and 17 m depths can range from 8.07 (*p*CO_2_ ∼ 246 µatm) to 7.87 (*p*CO_2_ ∼ 820 µatm) and from 7.80 (*p*CO_2_ ∼ 353 µatm) to 7.67 (*p*CO_2_ ∼ 1016 µatm), respectively [44]. Time-series data in the La Jolla kelp forest have also shown diurnal pH fluctuation ranging from 8.2 to 7.8 [44]. However, to date there are no reports of fish behavioural or physiological responses to current environmentally relevant CO_2_/pH variability (although [15] tested the effects of fluctuating CO_2_ from 450 to 2000 µatm in freshwater).

Similarly, most studies on the effects of OA on fish have so far focused on individual behaviour, yet roughly half of the world's fish species live in shoals for part of their life and about one-quarter of the world's fish species live in shoals for their entire life [46]. Shoaling is defined as any group of fish that remain together for social reasons [47,48]. Unlike schooling, shoaling fish do not necessarily display coordinated swimming [48]. Nonetheless, shoaling fish still benefit from enhanced predator detection [49], foraging [50] and social learning [51,52]. In the laboratory, shoaling behaviour has also been proposed as an index of anxiety (reviewed in [36]). These studies, and others in banded killifish (*Fundulus diaphanus*) [53], demonstrate that, in response to predatory cues or invasive stimuli, the shoal will become tighter (i.e. nearest neighbour distance (NND) and inter-individual distance (IID) will decrease). Conversely, pharmacological administration of anxiety-reducing (anxiolytic) drugs, some of which alter GABA_A­_ receptors, results in shoal dispersion (i.e. increased NND and IID) [54,55]. To the best of our knowledge, there have only been two studies on the effects of OA on shoaling behaviour: one reported a decreased tendency to associate with familiar shoal-mates in blue-green puller damselfish (*Chromis viridis*) [56], and the other described lower shoal cohesion in sand smelt (*Atherina presbyter*) [57].

Another understudied aspect of OA is the potential induction of differential effects on aquatic organisms during different seasons. If the effects of OA on fish behaviour were indeed caused by malfunction of neuronal functions as current models propose, such differential effects could exist due to differences in temperature, hormonal or nutritional status among other factors.

To answer some of these questions, we used blacksmith (*Chromis punctipinnis*), a damselfish found year-round in southern California waters [58,59]. Both juvenile and adult blacksmiths form loosely oriented shoals when unthreatened, and tighten into well-oriented shoals when threatened [58,59]. Juvenile blacksmiths typically live within 15 m of the surface, hiding in the kelp while feeding on the abundant plankton in the current [58]. Adult blacksmiths are found deeper in the water column, typically among rocky reefs and kelp forests [58,59].

Thus, the current study has the following objectives: (i) examine the impacts of future pH/CO_2_ levels on blacksmith individual behaviour; (ii) investigate the impacts of future pH/CO_2_ levels on blacksmith shoaling behaviour; (iii) compare the effects of oscillating acidification versus constant acidification on blacksmith individual and shoaling behaviour; and (iv) compare behavioural responses of blacksmiths collected and tested in different seasons of the year.

## 2. Material and methods

Juvenile blacksmiths were caught from drifting kelp paddies off the shores of La Jolla, adjacent to the Scripps Coastal Reserve (La Jolla, USA) in December 2014 and August 2015. Fish were collected and tested in two experimental sets to determine whether seasonal variability and/or water temperature may also affect blacksmith responses to CO_2_/pH. Blacksmiths from the same kelp paddies were habituated together within the Scripps Institution of Oceanography (SIO) flowing seawater aquarium for at least 30 days prior to experimentation. Blacksmiths were fed daily with live brine shrimp nauplii and frozen copepods. The initial behavioural measurements were performed in January 2015 (16.5 ± 0.1°C) on blacksmiths caught in December 2014. A subsequent set of individual behavioural measurements and a set of group behavioural measurements were performed in September 2015 (23.1 ± 0.1°C) on blacksmiths caught in August 2015. Blacksmiths were randomly selected from the holding tanks (January: *n* = 47; 3.2 ± 0.58 cm; 0.48 ± 0.02 g; September: *n* = 105; 3.3 ± 0.21 cm; 0.39 ± 0.01 g). There were no significant differences in length (January: *F*_2,44_ = 0.399; *p* = 0.674; September: *F*_2,44_ = 1.166; *p* = 0.321) or weight (January: *F*_2,48_ = 0.838; *p* = 0.439; September: *F*_2,48_ = 0.987; *p* = 0.380) within treatments. Blacksmiths were randomly assigned to control CO_2_/pH (January: *p*CO_2_ = 549 ± 5 µatm, 7.91 ± 0.00 pH units; September: *p*CO_2_ = 530 ± 3 µatm, 7.93 ± 0.00 pH units), constant acidification (January: *p*CO_2_ = 983 ± 19 µatm, 7.68 ± 0.01 pH units; September: *p*CO_2_ = 859 ± 9 µatm, 7.74 ± 0.01 pH units) and oscillating acidification (January: day: *p*CO_2_ = 587 ± 4 µatm, 7.89 ± 0.00 pH units; night: *p*CO_2_ = 1066 ± 66 µatm, 7.65 ± 0.03 pH units; September: day: *p*CO_2_ = 532 ± 4 µatm, 7.93 ± 0.00 pH units; night: *p*CO_2_ = 845 ± 2 µatm, 7.75 ± 0.00 pH units) treatments (table 1, electronic supplementary material, figure S1). Testing took place after 7 (individual behaviour) or 11 days (group behaviour) of exposure to control, constant or oscillating CO_2_ acidification. This time frame was chosen because it is consistent and comparable with our previous study on rockfish [14].

**Table 1.** Water chemistry during January 2015 and September 2015 experiments. pH_nbs_, alkalinity, salinity and temperature were measured as described in Material and methods. *p*CO_2_, Ω_aragonite_ and Ω_calcite_ were calculated using CO_2_SYS. Data presented as mean ± s.e.m.)

			oscillating acidification	
	control	constant acidification	day	night
*January 2015*				
pH_nbs_	7.91 ± 0.00	7.68 ± 0.01	7.89 ± 0.00	7.65 ± 0.03
alkalinity (µmol kgSW^−1^)	2234.04 ± 0.27	2232.81 ± 2.30	2233.99 ± 0.78	2233.70 ± 0.00
salinity (PSU)	33.4 ± 0.1	33.4 ± 0.1	33.4 ± 0.1	33.4 ± 0.1
*p*CO_2_ (µatm)	549 ± 5	983 ± 19	587 ± 4	1066 ± 66
Ω_aragonite_	1.90 ± 0.01	1.19 ± 0.02	1.80 ± 0.02	1.10 ± 0.06
Ω_calcite_	2.95 ± 0.02	1.85 ± 0.03	2.80 ± 0.02	1.70 ± 0.09
Temp (°C)	16.5 ± 0.1			
*September 2015*				
pH_nbs_	7.93 ± 0.00	7.74 ± 0.01	7.93 ± 0.00	7.75 ± 0.00
alkalinity (µmol kgSW^−1^)	2235.40 ± 2.02	2234.60 ± 1.33	2234.40 ± 1.85	2245.00 ± 0.00
salinity (PSU)	33.5 ± 0.3	33.5 ± 0.3	33.5 ± 0.3	33.5 ± 0.2
*p*CO_2_ (µatm)	530 ± 3	859 ± 9	532 ± 4	845 ± 2
Ω_aragonite_	2.48 ± 0.02	1.73 ± 0.02	2.50 ± 0.03	1.80 ± 0.01
Ω_calcite_	3.79 ± 0.04	2.64 ± 0.03	3.80 ± 0.04	2.70 ± 0.01
Temp (°C)	23.1 ± 0.1			

Seawater was continuously pumped from the Scripps Coastal Reserve into the header tanks, where the IKS Aquastar system (Karlsbad, Germany) monitored and recorded the temperature and pH values as well as manipulated the pH by bubbling CO_2_ gas into the three header tanks (electronic supplementary material, figure S1). The control header tank was not manipulated, and therefore reflected current local water conditions normally experienced by juvenile blacksmiths. Each header tank supplied water to three 20 l experimental tanks (0.3 l min^−1^) housing six blacksmiths each, and was covered with a transparent fibreglass lid to limit atmospheric exposure and slow CO_2_ degassing. Experimental tanks were randomly arranged and located on a shelf directly below the header tanks (see more details in ‘Data analysis’).

Additional pH measurements on header and experimental tanks were performed daily using a HACH portable pH probe (HQ40d with pH probe PHC101) to confirm proper electrode function and ensure that pH levels in the header tanks matched those in the animal tanks. Furthermore, discrete water samples were taken on the first, middle and last day of exposure and analysed for total alkalinity, salinity and pH in the Dickson laboratory (SIO; table 1). These discrete water samples were used to validate the IKS pH electrode measurements and calculate *p*CO_2_, Ω_aragonite_ and Ω_calcite_ using CO2SYS (v.2.1), with dissociation constants from Mehrbach *et al*. [60] as refitted by Dickson & Millero [61] (see also [62]) (table 1) [60–62]. Lighting was maintained in a 12 L : 12 D cycle using an automatic timer. In the oscillating acidification treatment, the IKS system triggered the CO_2_/pH switch two hours before the light/dark switch (electronic supplementary material, figure S1).

### 2.1. General behavioural testing protocol

All testing took place between the hours of 6.00 and 18.00. Recordings were captured by a FireWire 400 Color Industrial Camera with a Tamron CCTV lens (2.8–12 mm, f/1.4), and the videos were analysed with Ethovision XT motion tracking software system (v.10, Noldus, Leesburg, VA, USA). The arenas were placed in an enclosed testing chamber with even lighting. The arena was rotated 180° every four trials throughout the testing to compensate for any unintentional visual or auditory stimuli. All subjects were gently coaxed into a 500 ml container with their respective treatment water and transferred into a 30.5 × 15.3 cm area surrounded by white walls to allow the fish to acclimate to the conditions inside the testing chamber and arena. After 15 min, each blacksmith (or shoal of blacksmiths) was released into the centre of the testing arena. Testing began immediately afterwards and was recorded by an overhead camera. All blacksmiths were tested in their respective water treatment, and blacksmiths in the oscillating CO_2_/pH treatment were tested in control CO_2_/pH seawater as this was the condition experienced during the day, which was when behavioural testing took place. Blacksmiths were not fed for 24 h prior to behavioural testing [14]. Four blacksmiths from control, three from constant acidification and three from oscillating acidification treatments (all from different holding tanks) died during the exposure period from undetermined causes. Therefore, group behavioural testing was conducted with five blacksmiths in all cases.

### 2.2. Individual behaviour: light/dark preference test

The light/dark preference test was similar to earlier studies on zebrafish [63], splitnose rockfish [14] and black perch (*Embiotoca jacksoni*) [34]. The testing arena (30.5 cm × 15.3 cm × 19.0 cm) had black or white plastic walls (electronic supplementary material, figure S2), and was filled to a height of 7 cm with the respective treatment water. The bottom of the arena was white in both the light and dark zones to allow for motion tracking throughout the arena. The colour of the floor, white or black, has been previously shown to have no effect on light or dark zone preference in the light/dark preference test [63]. After seven days of treatment exposure, individual blacksmiths were placed into the arena for 15 min and the duration spent in the dark zone was quantified. Blacksmiths in the September experiment were returned to their experimental tanks for subsequent group behaviour testing.

### 2.3. Group behaviour: shoaling test

The shoaling test was based on previous studies on zebrafish [64,65]. The testing arena was a white, plastic, circular arena filled with 6 cm of the respective treatment water (electronic supplementary material, figure S2). After 11 days of treatment exposure, a shoal of five blacksmiths from the September experiment were simultaneously placed into the arena and recorded for 15 min. IID (the average distance of an individual fish to all other shoal mates) and time near the wall (thigmotaxis, 3.3 cm from the arena wall) were quantified. Each shoal of five blacksmiths constituted one sample, and a total of six shoals per treatment were analysed. The size of the thigmotaxic zone was based on the average body length of blacksmith (3.3 cm).

### 2.4. Group behaviour: novel object test

After 15 min of recording in the shoaling test (described above), a novel object was placed in the centre of the arena and fish behaviour was further recorded for another 15 min (electronic supplementary material, figure S2). The novel object was a multicoloured Lego figurine (5 cm tall) to avoid innate colour preference [14,15]. IID and time spent close to the novel object (both 3.3 and 6 cm radius) were analysed. These distances were chosen based on the average length of fish in this study and the radius used in previous novel object studies [14]. To the best of our knowledge, this was the first time the novel object test has been combined with a shoaling test. The rationale was that the novel object may be perceived as either predatory [66] or shelter-like stimuli [14], and this response may be altered by OA-like conditions.

### 2.5. Data analysis

Experimental tank effect was considered a random factor and nested in each treatment, which satisfies the experimental design guidelines proposed by Cornwall & Hurd [67]. Data were pooled when tank effect was determined to be conservatively not significant at *p* ≥ 0.250 (electronic supplementary material, table S1) [68]. The Shapiro–Wilk and Levene tests were used to evaluate the assumptions of normality and homogeneity (electronic supplementary material, table S1). Individual light/dark preference test behaviour was analysed using two-way analysis of variance (ANOVA) with month of the year (January, September) and pH treatments (control, constant acidification, oscillating acidification) as variables. One-sample *t*-tests were also used in the light/dark preference test parameters to compare time spent in a particular zone to 450 s (half of the duration of the test), as commonly used in these types of study [6,14,35]. Shoaling and novel object test behaviours were analysed with one-way ANOVA. All statistical analyses were completed using R (v. 0.98.1103) [69] and the R-package ‘car’ [70]. Unless otherwise noted, all values are given as mean ± s.e.m.

## 3. Results

### 3.1. Individual behaviour: light/dark preference test

**Figure 1.**
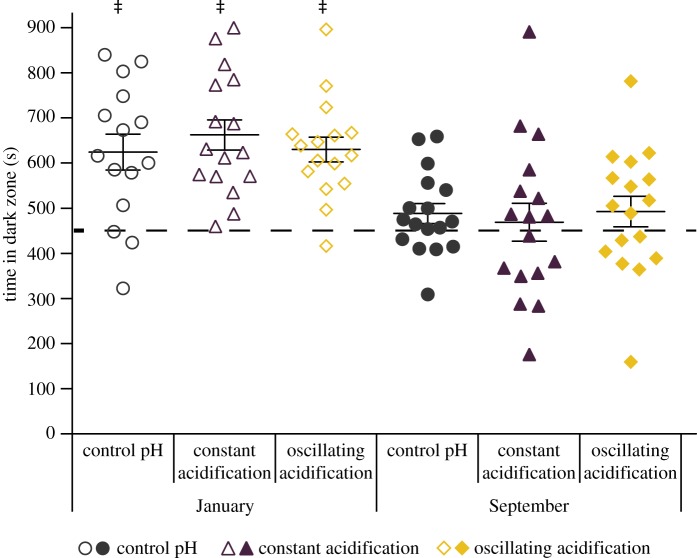
Control, constant acidification and oscillating acidification-exposed *Chromis punctipinnis* were individually placed in the light/dark preference test arena and their location recorded for 900 s. In January (*n* = 15–16), fish from all three treatments spent significantly more time in the dark zone than 450 s (‡*p* < 0.0001 in all cases), while in September (*n* = 17) they did not. Time spent in the dark zone was significantly affected by season (January versus September; *F*_1_,_92_ = 7.9540, *p* = 0.0059), but it was not significantly affected by CO_2_/pH treatments (*F*_2_,_92_ = 0.3557, *p* = 0.7017). There were no significant interaction effects (*F*_2_,_92_ = 0.4629, *p* = 0.6309). Data presented as mean ± s.e.m.

We first examined whether constant or oscillating acidification affected individual blacksmith light/dark preference (January: *n* = 15, 16, 16 and September: *n* = 17, 17, 17; control, constant acidification, oscillating acidification, respectively). Two-way ANOVA detected significant seasonal effect (January versus September experiments) on dark preference (*F*_1_,_92_ = 7.9540, *p* = 0.0059), but no significant effect of CO_2_/pH (control, constant acidification, oscillating acidification) (*F*_2_,_92_ = 0.3557, *p* = 0.7017) or interaction effect (*F*_2_,_92_ = 0.4629, *p* = 0.6309; figure 1). Similarly, one-sample *t*-test indicated that all three pH treatment groups in the January experiments spent significantly more than 450 s in the dark zone (*p* < 0.0001 for all three pH treatments), whereas none of the CO_2_/pH treatment groups in the September experiments had a significant preference for either the light or the dark zone (*p* = 0.1018, *p* = 0.6549, *p* = 0.2309 for control, constant acidification and oscillating acidification, respectively).

### 3.2. Group behaviour: shoaling test

**Figure 2.**
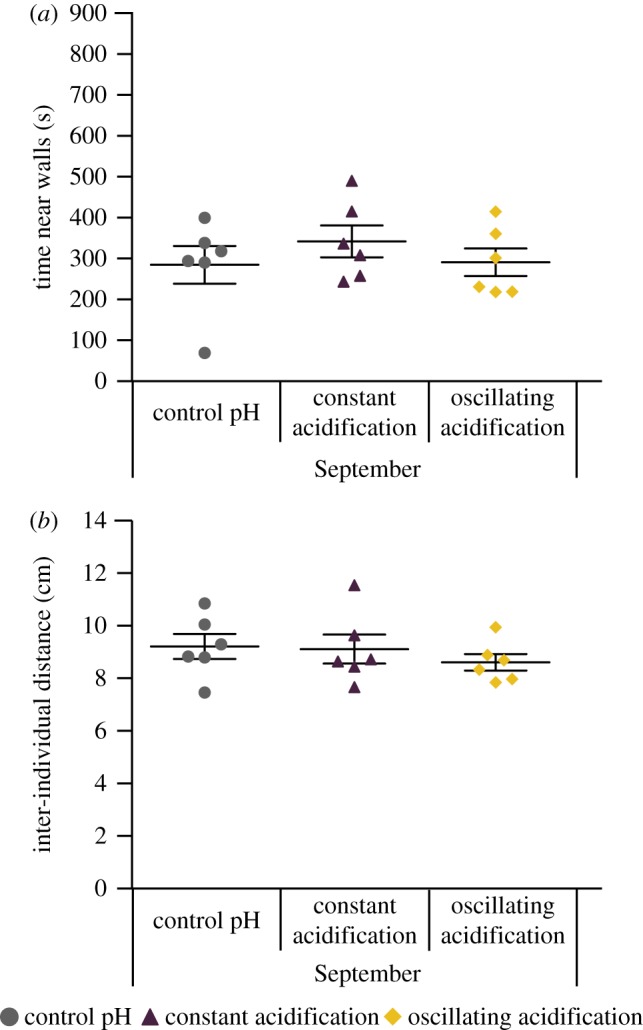
Control, constant acidification and oscillating acidification-exposed *Chromis punctipinnis* were placed into a shoaling test arena in groups of five and their locations recorded for 900 s (*n* = 6 shoals per treatment). (*a*) Time near walls was not affected by CO_2_/pH treatments (*F*_2,15_ = 0.621, *p* = 0.551). (*b*) Inter-individual distance was also not affected by CO_2_ treatments (*F*_2,15_ = 0.502, *p* = 0.615). Data presented as mean ± s.e.m. This test was only performed on the September groups.

We next examined whether constant or oscillating acidification affected blacksmith's shoaling behaviour (*n* = 6 shoals per treatment), with each data point representing a shoal of five fish. One-way ANOVA indicated no significant effect of constant or oscillating acidification on either thigmotaxis (*F*_2,15_ = 0.621, *p* = 0.551) or IID (*F*_2,15_ = 0.502, *p* = 0.615; figure 2).

### 3.3. Group behaviour: novel object test

**Figure 3.**
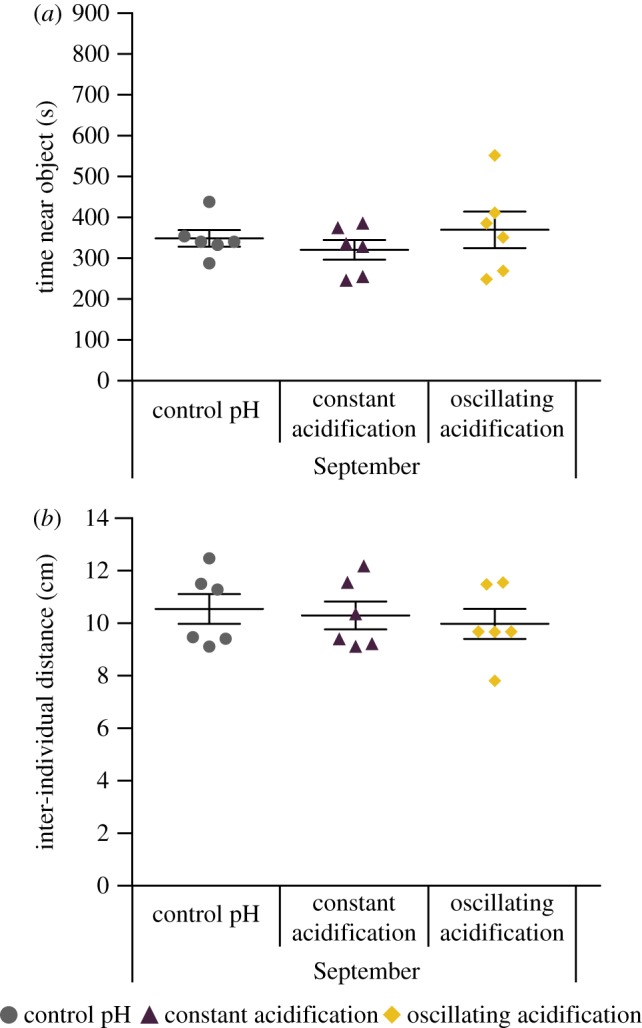
Immediately after the shoaling test, a novel object was placed into the arena and shoal behaviour was recorded for 900 s (*n* = 6 shoals per treatment). (*a*) Time near novel object was not affected by CO_2_/pH treatments (*F*_2,15_ = 0.159, *p* = 0.855). (*b*) Inter-individual distance to conspecifics was also not affected by CO_2_/pH treatments (*F*_2,15_ = 0.312, *p* = 0.737). Data presented as mean ± s.e.m. This test was only performed on the September groups.

Finally, we analysed the behaviour of a group of five blacksmiths after the introduction of a novel object (*n* = 6 shoals per treatment). One-way ANOVA found no significant differences in constant or oscillating acidification in either time near the object (6 cm radius; *F*_2,15_ = 0.159, *p* = 0.855) or IID (*F*_2,15_ = 0.312, *p* = 0.737; figure 3). There were also no significant differences (*F*_2,15_ = 0.283, *p* = 0.758) when a 3.3 cm radius was used to analyse time near the object.

## 4. Discussion

A common limitation of laboratory studies is that the experimental conditions do not mimic the conditions experienced by fishes in the wild. With this in mind, we added two variables not commonly implemented in OA studies; oscillating acidification to replicate the natural variability encountered in kelp forests and seagrass beds, and testing of group behaviour to examine the natural social behaviour that is observed in most fish species. However, neither individual nor group behaviour were affected by either constant or oscillating acidification, suggesting that blacksmiths are resilient to environmental CO_2_/pH variability and to near-future OA scenarios, at least in relation to the parameters tested. On the contrary, the season in which blacksmiths were caught and tested did affect individual blacksmith behaviour, which warrants further investigation.

The lack of effect of OA conditions on blacksmith light/dark preference is in contrast with our earlier study on splitnose rockfish, in which exposure to constant acidification induced a significant dark preference [14]. The aforementioned rockfish study and the current blacksmith study are comparable, because both collected fish from drifting kelp paddies offshore of La Jolla, California, exposed them to similar CO_2_/pH conditions and quantified fish behaviour using the same light/dark preference test. Constant and oscillating acidification also did not affect the group behaviour parameters tested on blacksmith in this study. These results have important caveats. Firstly, although the means of IID and thigmotaxis from control and treatments were not statistically different from each other and there were no obvious trends, the low number of replicates (*n* = 6) affects the statistical power. However, because each replicate was a shoal of five fish, and because there were three experimental conditions, our sample size of *n* = 6 for each condition involved the use of 90 fish. Thus, increasing the number of replicates represents a challenge both in terms of infrastructure and testing logistics. Additionally, the lack of information about blacksmith group behaviour and relevant experimental tests makes it difficult to assess the relevance of the results for real-world conditions. These limitations clearly indicate that there is a need for more research and resources dedicated to OA studies.

One mainstream hypothesis behind OA-induced behavioural changes in fish is altered intracellular/extracellular bicarbonate and chloride gradients which change GABA_A_ receptor function as a side effect of blood acid–base regulation [21,40,41] (reviewed in [22]). As fish accumulate $\text{HC}{{\text{O}}_{3}}^{-}$ to buffer blood pH, Cl^−^ concentration is proposed to be reduced in an equimolar amount, and reverse the flux of Cl^−^ across GABA_A_ receptors [21]. Thus, GABA-induced GABA_A_ receptor opening could result in neuronal depolarization instead of hyperpolarization, which could explain the alteration in behaviours.

Multiple studies have reported alterations in fish anxiety-like behaviour in response to elevated CO_2_ levels. In addition to the above-mentioned increased anxiety in rockfish [14], OA-exposed marine three-spined stickleback (*Gasterosteus aculeatus*) spent significantly less time near the novel object, similarly suggesting increased anxiety [10]. CO_2_ acidification also induced an alteration in anxiety-like behaviour in pink salmon in freshwater; however, in this case, anxiety was decreased in the novel-approach test [15]. However, exposure to elevated CO_2_ levels did not affect the behaviour of several other fish species subjected to similar tests. For example, OA-like conditions did not significantly affect predator avoidance [23], behavioural lateralization or boldness [24] in juvenile Atlantic cod (*Gadus morhua*), and it also did not affect anxiety-like behaviour in red drum (*Sciaenops ocellatus*) [25].

These species-specific differences have been attributed to a variety of potential causes, from different iono-regulatory mechanisms and capacity, to different life-history traits [15], such as living in a marine, estuarine or freshwater environment, living in the open ocean, kelp forest, mangrove or reef, or being a migratory species (reviewed in [41]). The lack of effect of OA on blacksmith behaviour in the current study could be due to a variety of not mutually exclusive reasons. For example, blacksmiths may be able to regulate the acid/base status of their internal fluids, so OA-relevant elevations in CO_2_ levels do not affect neuronal function, or they may be able to regulate neuronal membrane potential to offset potential effects of OA on the chemistry of their internal fluids (reviewed in [41]). Exploring these mechanisms in fish species whose behaviour is altered by OA-like conditions, and comparing them to species that are resilient could provide valuable insights on the potential effects of OA on fish in the wild. However, this is not a trivial task, as it requires measuring the concentrations of $\text{HC}{{\text{O}}_{3}}^{-}$ and Cl^−^ inside neurons and in cerebrospinal fluid. This has never been done in any fish, and is extremely challenging because the putative changes in ion concentrations are small and likely to be affected by the sampling procedures (reviewed in [41]).

Another interesting and unexpected finding from our study was the significantly different anxiety-like behaviour measured in both control and OA conditions in experiments in January (winter) compared to the experiments in September (late summer). Both sets of experiments were conducted under identical illumination and diet conditions; however, seawater temperature was approximately 6.50°C higher in the summer. Thus, the difference in dark preference between winter and summer could have been due to temperature. However, several other potentially relevant parameters were not measured, such as hormonal levels, and cannot be ruled out. Regardless, the observed differences in control behaviours between the two seasons strengthens the finding that the OA-like conditions tested here have no effect on blacksmith because there was no effect of OA on anxiety-like behaviour in either season. However, effects of OA on other aspects of blacksmith behaviour, most notably olfactory discrimination, have not been investigated here and will constitute the basis for future research.

Blacksmith responses in the current study also differed from studies on tropical damselfish that have found a variety of behavioural impairments in response to OA-like conditions, including altered behavioural lateralization (yellowtail demoiselle, *Neopomacentrus azysron*) [21], altered olfactory abilities (*Pomacentrus wardi* [18]; *Dascyllus aruanus* and *P. moluccensis* [71]) and impaired learning of olfactory cues (*P. amboinensis* [12,13]). Interestingly, the ability to sense anti-predator cues after OA-like treatments was considerably different in four congeneric species of damselfish (*P. moluccensis, P. amboinensis, P. nagasakiensis and P. chrysurus*) [72]. Because these fish shared similar ecology and life history, the authors concluded that their differential resilience to OA-like conditions may be due to unidentified physiological differences [72]. The differential response to OA-like conditions between blacksmith (a temperate damselfish) and tropical damselfishes may be explained by the same rationale. However, variability in life stages and ecological habitats make a direct comparison to our study difficult. Furthermore, performing light/dark and shoaling tests on tropical damselfish species, or olfactory discrimination tests on blacksmith, is necessary for direct comparisons between species.

In summary, the behaviour of juvenile blacksmith was not affected by CO_2_-acidified conditions previously demonstrated to affect juveniles of a sympatric rockfish species. These results suggest these two fish species are not equally susceptible to the predicted future OA, and identify blacksmith and splitnose rockfish as a potential ‘winner’ and ‘loser’ species, respectively. However, this possibility should be experimentally confirmed by other behavioural and physiological tests, which would ideally combine laboratory and field studies.

## Supplementary Material

Supplementary Table 1. One-way ANOVA testing the random effect of animal holding tank nested within treatment.

## Supplementary Material

pH values in the experimental treatments

## Supplementary Material

Schematic of the testing arena used in the behavioural tests

## Data accessibility

The datasets supporting this article are presented in the text, table, figures and electronic supplementary material.
