# Supplementary material for: CO2-induced ocean acidification does not affect individual or group behaviour in a temperate damselfish
Source: R Soc Open Sci. 2017 Jul 5;4(7):170283. doi: 10.1098/rsos.170283 (PMC5541549; doi:10.1098/rsos.170283)
Supplement: Supplementary Table 1. One-way ANOVA testing the random effect of animal holding tank nested within treatment. [file rsos170283supp1.docx]

**Supplementary Table 1.** One-way ANOVA testing the random effect of animal holding tank nested within treatment. All parameters reported *p*-values above 0.25, a conservative significance level recommended by Quinn and Keough, 2002, and therefore removed tank as a random factor and the data pooled for analysis. Shaprio-Wilks test and Levene’s test were used to assess the assumptions of normality and homoscedasticity, respectively.

| **parameter** | **tank effect** | | **Shapiro-Wilks test** | **Levene’s test** |
| --- | --- | --- | --- | --- |
|  | **F Score** | ***p*-value** | ***p*-value** | ***p*-value** |
| Light/Dark Preference Test: Time in Dark (Jan) | 0.093 | 0.912 | 0.8045 | 0.3379 |
| Light/Dark Preference Test: Time in Dark (Sep) | 1.389 | 0.260 | 0.5925 | 0.1270 |
| Shoaling Test: Time near Walls | 0.842 | 0.455 | 0.7310 | 0.9904 |
| Shoaling Test: Inter-Individual Distance | 0.024 | 0.976 | 0.2382 | 0.7672 |
| Novel Object Test: Inter-Individual Distance | 0.038 | 0.963 | 0.0994 | 0.8193 |
| Novel Object Test: Time near Object | 0.072 | 0.931 | 0.1829 | 0.2101 |
