# Supplementary figures and images for: CO2-induced ocean acidification does not affect individual or group behaviour in a temperate damselfish
Source: R Soc Open Sci. 2017 Jul 5;4(7):170283. doi: 10.1098/rsos.170283 (PMC5541549; doi:10.1098/rsos.170283)

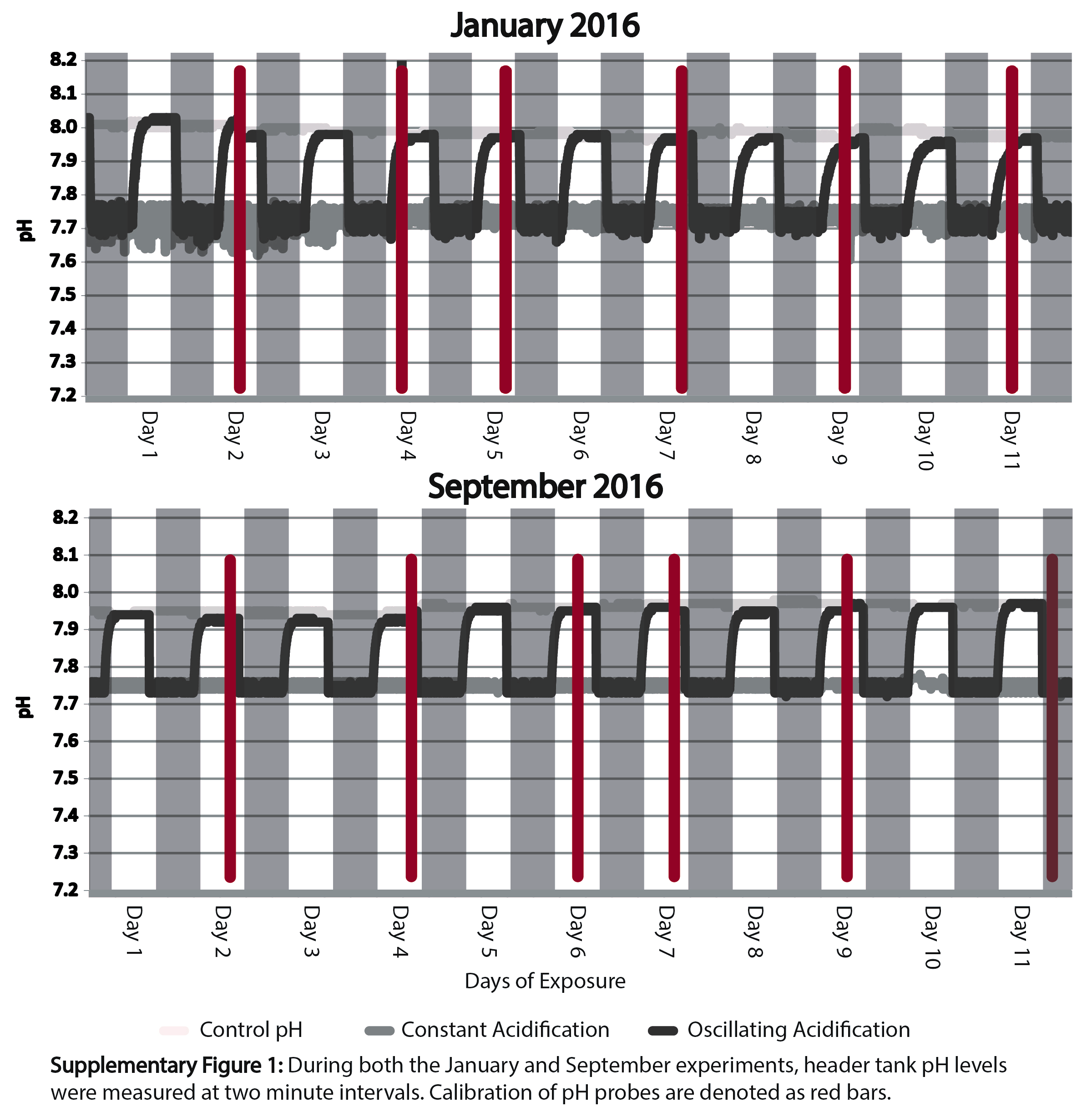

Supplement: pH values in the experimental treatments [file rsos170283supp2.tif]

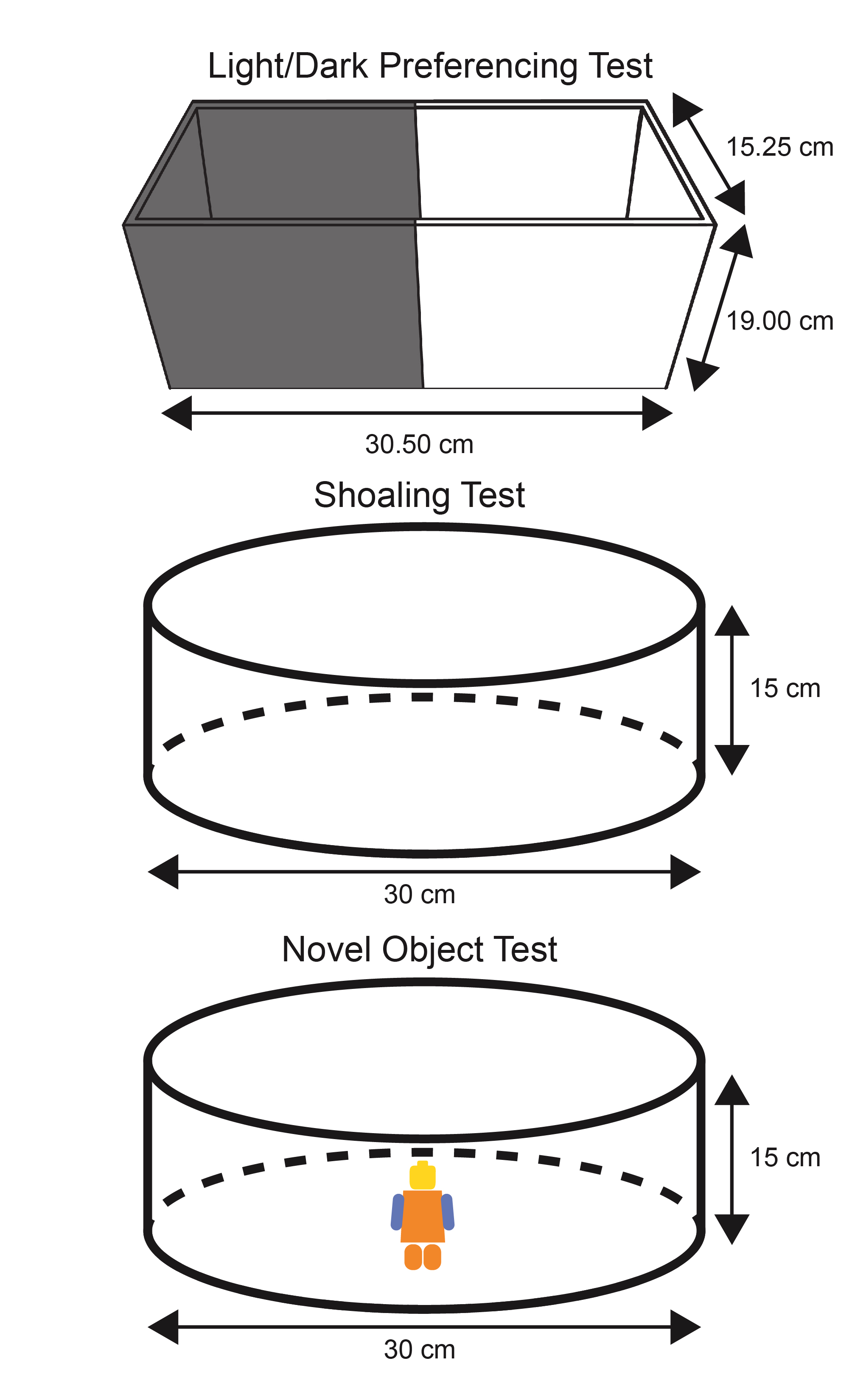

Supplement: Schematic of the testing arena used in the behavioural tests [file rsos170283supp3.jpg]
